# Differences in COVID-19 vaccine acceptance and uptake according to region of birth: findings from a cross-sectional survey in Sweden

**DOI:** 10.3389/fpubh.2026.1861648

**Published:** 2026-06-24

**Authors:** Mariam Hassan, Adam Mitchell, Malin Inghammar, Jonas Björk, Louise Bennet

**Affiliations:** 1 Department of Clinical Sciences Malmö, Family Medicine and Community Medicine, Lund University, Lund, Sweden; 2 Department of Laboratory Medicine, Division of Occupational and Environmental Medicine, Lund University, Lund, Sweden; 3 Department of Clinical Sciences Lund, Division of Infection Medicine, Lund University, Lund, Sweden; 4 Department of Infection Prevention and Control, Region Skåne, Diseases, Skåne University Hospital, Lund, Sweden; 5 Clinical Studies Sweden, Forum Söder, Skåne University Hospital, Lund, Sweden

**Keywords:** behavioural drivers, social drivers, vaccine acceptance, vaccine hesitancy, vaccine uptake

## Abstract

**Introduction:**

High vaccine uptake was crucial in preventing morbidity and mortality during the COVID-19 pandemic. The study aimed to investigate barriers and drivers of vaccination in Sweden across birth region groups with varying COVID-19 vaccine uptake.

**Methods:**

A cross-sectional anonymous survey was distributed non-randomly among individuals born in Sweden and immigrants from Western and non-Western countries (*n* = 3,281). Six domains, ‘trust in vaccines’, ‘benefit–risk balance’, ‘trust in institutions’, ‘injunctive norms’, ‘descriptive norms’, and ‘accessibility’ were generated, with vaccine acceptance scores (range 1–5), and summary vaccine scores (range 6–30). Linear regression analysis was performed to investigate the associations between the summary acceptance score and vaccine uptake, overall and by region of birth. Associations between the specific domains and vaccine uptake were modelled using logistic regression (odds ratios (OR) and 95% confidence intervals (CI)), stratified by region of birth.

**Results:**

The associations between vaccine acceptance and uptake were generally strong, but weaker among individuals from non-Western countries. Vaccinated individuals from Sweden and Western countries had 9.5 (95% CI: 9.2–9.7) and 8.9 (95% CI: 8.1–9.7), respectively, higher average vaccine acceptance score than their unvaccinated counterparts, compared with only 2.8 (95% CI: 2.0–3.6) higher average score among vaccinated individuals from non-Western countries. Among individuals from Sweden and Western countries, ‘descriptive norms’ (OR = 2.6; 95% CI: 2.0–3.4; and OR = 3.6; 95% CI: 1.5–8.7, respectively) and ‘trust in vaccines’ (OR = 2.5; 95% CI: 1.9–3.2; and OR = 4.2; 95% CI: 2.0–8.8, respectively) had the strongest associations with vaccine uptake. Among individuals from non-Western countries, associations with vaccine uptake were only observed for ‘trust in vaccines’ (OR = 1.6; 95% CI: 1.1–2.3) and ‘benefit–risk balance’ (OR = 1.7; 95% CI: 1.2–2.4).

**Conclusion:**

There were significant differences across region groups in the impact of BeSD factors on vaccination. The weaker associations observed among individuals from non-Western countries suggest that these factors may not explain vaccine uptake in this group, where vaccine acceptance was moderately high regardless of vaccination status.

## Introduction

The COVID-19 pandemic impacted population health across the globe, affecting an estimated 778 million persons, including 7.5 million deaths to date in 2025 (1). In this context, the role of immunisation became essential to prevent morbidity and mortality. In Sweden, the vaccine uptake against COVID-19 was generally high, with over 80% of the adult population receiving at least two doses in 2022 (2). However, variations in vaccine uptake have been identified (3, 4), potentially creating ‘pockets’ of low vaccine uptake in society. Previous literature has pointed towards an unequal burden of COVID-19-related morbidity and mortality among minoritised ethnic groups and groups with low socioeconomic position, both globally and in Sweden (5, 6). This burden could partly be explained by the lower vaccine uptake among these groups, observed across the different phases of vaccine rollout in Sweden, where priority groups shifted throughout the phases (7–10). Nonetheless, suboptimal vaccine uptake was seemingly not isolated to one population group (7, 9). A systematic review focusing on studies internationally (11), found that both high and low socioeconomic positions have been associated with barriers to vaccination (11). Similarly, reports of barriers to vaccination by race/ethnicity/racialisation in the United States of America have been mixed [e.g., (12–16)], further strengthening the notion that factors influencing vaccine uptake vary by context.

The factors impacting vaccination have been conceptualised under the term ‘vaccine hesitancy’ (11). In the present study, vaccine hesitancy was defined as a motivational state of ambivalence or opposition towards vaccination (17). Vaccine hesitancy is encompassed by an array of interconnected decision-making attitudes (18, 19) shaped by the social-political environment, and conditions each other and vaccine uptake to varying degrees. Vaccine hesitancy includes perceived disease risks and vaccine trust (20), social norms (21), health literacy (22, 23), accessibility to healthcare services (24), and recommendations from and trust in healthcare professionals (21, 25). While considerable attention has been given to what factors influence vaccine uptake, there has been less focus on the differences in the importance of these factors across social population groups. Thus, further research is warranted to elucidate the context-specific barriers and drivers to vaccination, with special attention to potential differences and similarities across minority groups, in this study defined as region of birth. These categorisations were used in this study to analyse health and healthcare access inequalities related to social stratification, specifically of the dimensions of socioeconomic position and immigration, as differences related to these dimensions have been observed in healthcare utilisation, for instance, screening programmes, and COVID-19 morbidity and mortality (10, 26–28). This study aimed to investigate barriers and drivers of vaccination among various social population groups with suboptimal COVID-19 vaccine uptake.

## Methods and statistical analyses

### Study design and study population

This cross-sectional study was based on responses from an anonymous survey distributed non-randomly through a combination of volunteer and purposive sampling in Skåne (Scania County in Southern Sweden with 1.4 million residents in 2024; mean age 42 years, 51% women). Around 25% are born abroad (16% outside of the European Union), 20% live in areas with socioeconomic challenges in 2023, and these areas tend to have a multiethnic population (29–31). The distribution of the survey generated a sample size of 3,281 participants. This sampling strategy was used in response to a previous conventional non-anonymous epidemiological design using random sampling from population registers stratified by residential area and vaccination status, where unvaccinated individuals, immigrant groups, and individuals with low socioeconomic position were underrepresented among participants. Therefore, the recruitment strategy in the current study was designed to improve participation among the observed underrepresented groups, including distributing it anonymously to increase participation of unvaccinated individuals. The questionnaire was administered on social media platforms, using volunteer sampling. To reach immigrant groups and individuals with low socioeconomic position to a greater extent, it was supplemented with a purposive sampling strategy, where the questionnaire was distributed in local settings. The purposive sampling strategy was used in parallel with the volunteer sampling on social media platforms in the year 2023. However, to further improve the participation rate among immigrant groups, we decided to only use the purposive sampling strategy in 2024. These recruitment strategies do not provide information on non-participation. We therefore compared the sociodemographic information of the sample with that of the underlying background population of Skåne using register data from the Regional Population Register, the longitudinal integrated database for health insurance and market studies, and the National Vaccination register.

The questionnaire was administered during the spring of 2023 (May 30 to July 10) and 2024 (May 20 to July 31). The questionnaire was disseminated digitally on social media in Skåne, Sweden, on the Medical Faculty at Lund University’s Facebook page, on X (formerly Twitter), and among various associations’ WhatsApp groups to reach a broad and local audience. Furthermore, the questionnaire was distributed via flyers containing an online link and a quick response code at libraries, folk high schools (*folkhögskolor*), university networks, and local stores. In total, *n* = 2,805 digital responses were obtained. We also obtained *n* = 476 responses completed in paper form, distributed by authors LB and MH and a research assistant in different neighbourhood areas, in Swedish for immigrants classes, associations, libraries, local stores, cafés, local community events, in primary health clinics, and among healthcare professionals providing health and social care to older adults and their networks in Skåne. The survey was available in Swedish, English, Arabic, Somali, Ukrainian, and Russian in both digital and paper form.

### Survey questionnaire

The design of the anonymous questionnaire was based on the survey developed by the World Health Organization using the Behavioural and Social Drivers (BeSD) framework (17, 32). We have previously utilised this questionnaire (33). However, in the current study, sociodemographic questions were added because linkage to registers was not possible due to the questionnaire being anonymous. The questionnaire was adapted to the Swedish context to ensure the relevance of the posed questions (33). The survey included questions on sociodemographic information, vaccine behaviour, attitudes, and factors influencing the intention and decision to get vaccinated based on the four BeSD core domains (17, 32, 34) represented by (1) thoughts and feelings about vaccines; (2) social processes that drive or inhibit vaccination; (3) motivation or hesitancy to seek vaccination; and (4) practical issues involved in seeking and receiving vaccination (17).

**Figure 1:**
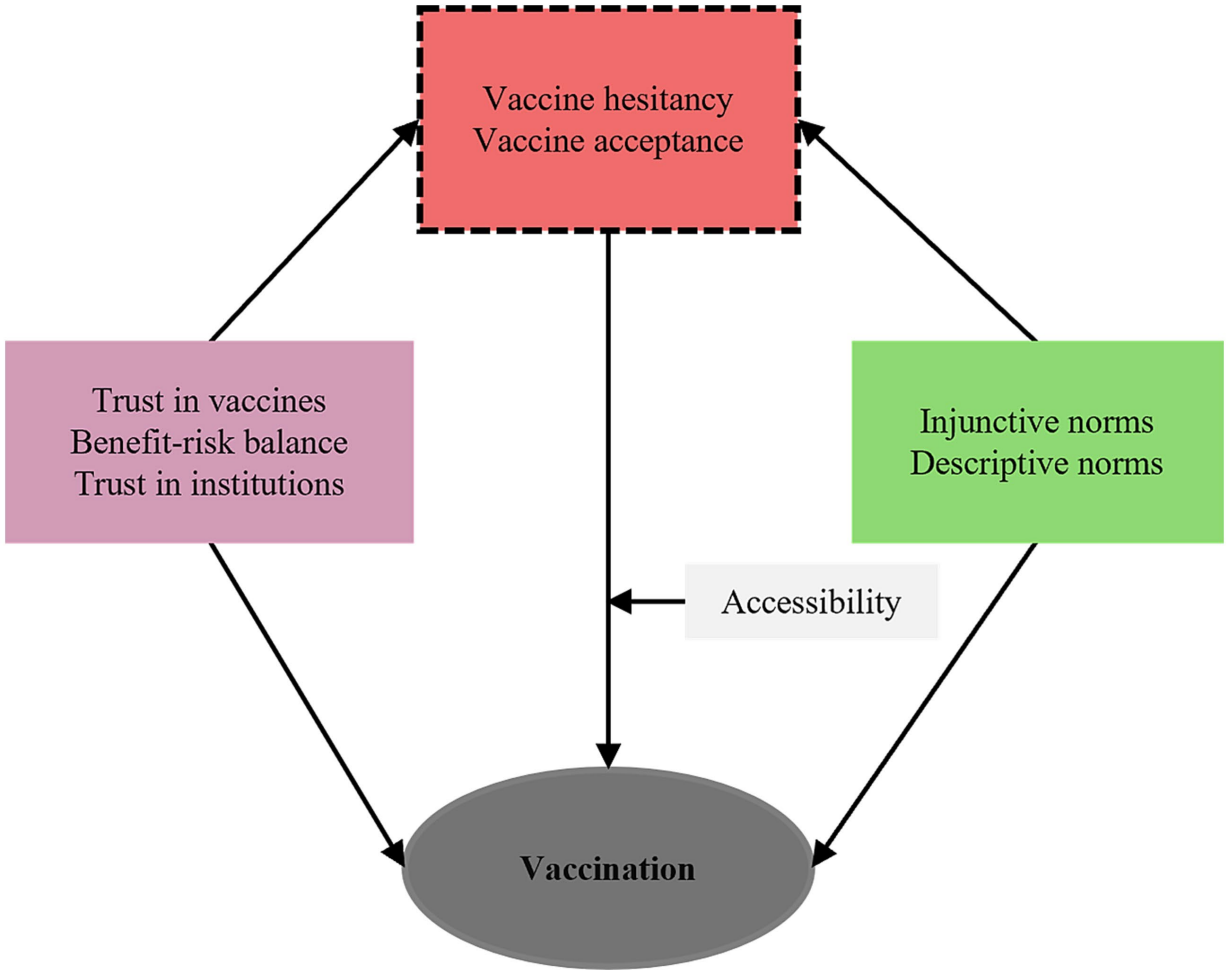
Conceptual framework based on the world health organization’s behavioural and social drivers (BeSD) of vaccination framework for COVID-19 vaccinations (17). The domains ‘trust in vaccines’, ‘benefit–risk balance’, ‘trust in institutions’, ‘injunctive norms’, ‘descriptive norms’, and ‘accessibility’, as well as the outcome defined as ‘vaccine uptake’, were the domains we defined and created in this study (33).

Based on the BeSD framework and its core domains (17), we created six domains to obtain a nuanced understanding of factors influencing vaccine decisions, allowing future interventions to be more effectively tailored to promote vaccine uptake (Figure 1). The domains ‘trust in vaccines’ (e.g., ‘*I have seen or heard bad things about COVID-19 vaccine*’), ‘benefit–risk balance’ (e.g., *‘I have a fear of vaccine side effects’*), and ‘trust in institutions’ (e.g., *‘confidence in healthcare’*) constituted together various aspects of the BeSD core domain ‘thinking and feeling’. Similarly, the domains in our study, ‘injunctive norms’ (e.g., *‘the recommendations of the Public Health Agency of Sweden are important for my position on vaccinations’*), and ‘descriptive norms’ (e.g., *‘in the context I live in, it is important to get vaccinated’*), constituted the core domain ‘social processes’ in the BeSD framework. Furthermore, the core domain ‘practical issues’ in the BeSD framework corresponded to the domain ‘accessibility’ (e.g., *‘important that there are short waiting times for vaccination’*) in this study. These domains were theoretically considered to lead to the core domain ‘motivation’, a latent variable, which may either lead to vaccination or no vaccination.

### Variables

#### Survey questionnaire

##### Vaccine uptake

Based on the questions ‘*Are you vaccinated against COVID-19?*’ and ‘*If you have been vaccinated against COVID-19, how many doses have you received?*’, vaccine uptake (yes/no) was defined as ‘yes’ if the individuals had responded that they were vaccinated or had received at least one vaccine dose to the former questions.

##### Vaccine acceptance scores

In line with our description in a previous study (33), the six domains were based on several questions, each with a 4 or 5-point Likert scale. The responses for each question in every domain were dichotomised into agreeing (1 point) or disagreeing (0 points) responses. In instances where responses agreeing with an item indicated distrust or risks rather than trust and benefits of vaccination, the scoring was reversed so that higher scores consistently reflected vaccine acceptance (Supplementary Table 1 for the scoring and the specific questions within the domains). To ensure aggregation of agreeing or disagreeing results, individuals who responded ‘no opinion’ were coded as missing. From the non-missing responses, the aforementioned six domains were created, summing and rounding the scores to the nearest integer, creating a vaccine acceptance score. Each domain contained a combination of questions covering themes of ‘trust in vaccines’, ‘benefit–risk balance’, ‘trust in institutions’, ‘injunctive norms’, ‘descriptive norms’, and ‘accessibility’. The proportion of agreeing responses in each domain was converted to a score between 1 and 5, where higher scores indicated greater levels of vaccine acceptance:


Proportion of agreeing responses×4+1

Individuals did not obtain a score for a specific domain if they had missing answers on at least two questions within a domain. A summary vaccine score across the six domains was calculated from the number of valid scores to ensure a range from 6 to 30:


$$\frac{\mathrm{Sum}\;\text{of valid domain scores}}{\text{Number of valid domain scores}}\times 6$$

Individuals who had missing values across at least four domains were not given a summary score.

Cronbach’s Alpha was calculated for each domain and for the sum score as a measure of internal consistency within each domain and for the sum score. The Cronbach’s Alpha value for the summary vaccine acceptance score was 0.87, indicating a good internal consistency for the overall scale (Supplementary Table 2). Reliability coefficients for the domains separately ranged from 0.60 to 0.79, demonstrating moderate and high internal consistency across the domains. This is in line with our previous study developing the vaccine acceptance scoring (33).

#### Explanatory variables

Individuals were categorised by region of birth (‘Sweden’/‘Western countries’/‘non-Western countries’). Individuals born in Sweden were categorised as ‘Sweden’. Individuals born in the Nordic countries, Europe, North America, and Oceania, excluding Sweden, were categorised as ‘Western countries’. Individuals born in the Middle East/Southwest Asia, Asia, Africa, and South America were categorised as ‘non-Western countries’. For simplicity, regions of birth groups will be referred to as region groups throughout the paper. Detailed self-reported data on sociodemography and health were also included and used to categorise the following variables. Gender/sex was defined as ‘men’/‘women’. Age was categorised into three groups, namely ‘18–49’, ‘50–64’, and ‘65+’. To avoid unnecessary reduction in sample size and to retain statistical power, participants from non-Western countries with missing values in the age variable were categorised into the 18–49 age group. This decision was informed by the demographic characteristics of the recruitment settings, where the general age was younger than 50, indicating a predominantly younger population. Individuals with missing age values in the other region groups were treated as missing and not reassigned to alternative categories. Civil status was included as a binary variable (‘single’/‘not single’). Education was categorised into three groups, namely, ‘low-’, ‘middle-’, and ‘high educational level’. ‘Low educational level’ consisted of individuals reporting primary education or less. The ‘middle educational level’ included short- and long secondary education, and folk high school. A ‘high educational level’ included tertiary education. Financial hardships based on the question ‘*How often have you during the past 12 months had difficulties paying your bills (rent, electricity, telephone, interests, amortisation, insurance etc?)*’ indicated an individual’s socioeconomic position together with education. Financial hardships were dichotomised, and individuals who responded, ‘every month’, ‘half of the year’s months’, and ‘once in a while’ were categorised as having ‘financial hardships’. Individuals who responded ‘never’ were categorised as having ‘no financial hardship’. Furthermore, comorbidities were defined based on questions about cardiovascular disease, diabetes, high blood pressure, lung diseases such as asthma or chronic obstructive pulmonary disease, or other diseases. Individuals who responded yes to having comorbidities were categorised as such. The rest of the respondents were categorised as having ‘no comorbidities’.

### Register data

#### Variables

All the variables used from the registers were from the end of the year 2020, except for information on vaccine uptake (defined as individuals with at least one COVID-19 vaccine dose received) and age, which were from early 2024 and the end of the year 2023, respectively (approximately in the middle of the recruitment period for survey participants). Vaccine uptake was defined as individuals receiving at least one COVID-19 vaccine dose by the end of the year 2023. For the categorisation of the region groups, we constructed a variable reflecting the individuals who participated in the survey. Therefore, Swedish born individuals were those born in Sweden. Western countries were defined as individuals born in Europe because individuals categorised as Western in our survey sample were predominantly born in European countries. This included all current European countries, as well as former countries that have since dissolved. Non-Western countries were defined as Arabic-speaking countries in the Middle East/Southwest Asia, and Somalia, reflecting that respondents in this category in our sample were overwhelmingly born in this region and country. Regions not represented in the survey sample were excluded from the register data to ensure a valid comparison between the sample and the population. Gender was binary and defined as ‘men’/‘women’. Age was categorised into three groups, namely ‘18–49’, ‘50–64’, and ‘65+’, and everyone residing in Skåne younger than 18 years was excluded. Furthermore, married individuals and individuals with a registered partnership were considered ‘not single’, while widows/widowers, surviving registered partners, divorced, and separated partnerships were considered ‘single’. Educational level was categorised into three groups, where ‘low educational level’ consisted of individuals with registered primary education or less. The ‘middle educational level’ included short- and long secondary education, and a ‘high educational level’ included tertiary education.

### Statistical analyses

Besides descriptive statistics of the underlying population and the sample, a linear regression analysis was performed to investigate the associations between the summary vaccine acceptance score, as the outcome variable, and vaccine uptake and region groups, while adjusting for between-group differences in age, sex/gender, educational level, financial hardships, civil status, and comorbidities. Standard procedures for checking linear regression assumptions were performed, which included assessing homoscedasticity, residual normality, and multicollinearity, and evaluating robustness by comparing regular standard errors and confidence intervals with those based on robust standard errors. Potential effect modification between region groups and vaccine uptake was investigated by including cross-product interaction terms. Furthermore, to assess potential differences in associations between vaccine uptake and the vaccine acceptance domains, vaccine uptake was modelled using logistic regression analysis with each domain included simultaneously (multidomain model), both unadjusted and adjusted by the aforementioned variables, stratified by region groups. Unadjusted and adjusted models for each domain separately (single domain models) were also reported. The linearity of the logit assumption was also assessed before analysis. All analyses were performed using complete-case analysis based on the predefined variable specifications, restricting the sample to individuals with complete data on all variables included in the model. All statistical analyses were conducted in Stata SE 19.0.

## Results

### Sample characteristics

Generally, across all region groups, our sample included more women, individuals aged 50–64 years, and individuals who were in partnership, i.e., ‘not single’, compared to the corresponding population subgroups of Skåne (Supplementary Table 3). Furthermore, fewer individuals were older than 65 years in our sample compared to the population subgroups of Skåne. Among individuals from Sweden and Western countries, our sample had a greater proportion of individuals with a high educational level. However, there were more individuals with a middle educational level among the group from non-Western countries in our sample compared to the corresponding group in the population. In the sample, there were more unvaccinated individuals among individuals from Sweden and Western countries, while the opposite was observed among individuals from non-Western countries.

In total, 3,281 individuals responded to the survey (2,481 were from Sweden, 312 were from Western countries, and 416 were from non-Western countries; Table 1). Proportions of civil status, and comorbidities were similar across all region groups. Vaccinated and unvaccinated generally had similar sociodemography within each of the three region groups. Comorbidities were consistently more common among vaccinated than unvaccinated individuals (Supplementary Table 4). Generally, individuals responding to the questionnaire digitally were recruited using volunteer sampling, while individuals responding to the questionnaire in paper form were recruited using purposive sampling.

**Table 1:** Descriptive characteristics of the participants in the full sample and stratified by region of birth groups.

	Full sample (*n* = 3,281)	Sweden (*n* = 2,481)	Western countries (*n* = 312)	non-Western countries (*n* = 416)
Mode of administration of questionnaires by year				
Year 2023				
Digital	2,795 (97.0%)	2,450 (99.9%)	265 (100.0%)	47 (38.5%)
Paper form	85 (3.0%)	3 (0.1%)	0 (0.0%)	75 (61.5%)
Year 2024				
Digital	10 (2.5%)	1 (3.6%)	1 (2.1%)	8 (2.7%)
Paper form	391 (97.5%)	27 (96.4%)	46 (97.9%)	286 (97.3%)
Age				
18–49	1,371 (43.8%)	972 (40.0%)	137 (45.1%)	238 (66.3%)
50–64	1,216 (38.8%)	975 (40.2%)	125 (41.1%)	107 (29.8%)
65+	546 (17.4%)	480 (19.8%)	42 (13.8%)	14 (3.9%)
Missing	148	54	8	57
Sex/gender				
Women	2,270 (71.7%)	1,723 (71.4%)	227 (73.9%)	292 (71.6%)
Men	896 (28.3%)	690 (28.6%)	80 (26.1%)	116 (28.4%)
Missing	115	68	5	8
Civil status				
Single	963 (30.0%)	738 (29.9%)	87 (28.2%)	126 (31.9%)
Not single	2,243 (70.0%)	1,732 (70.1%)	221 (71.8%)	269 (68.1%)
Missing	75	11	4	21
Education				
Low educational level	197 (6.1%)	83 (3.4%)	10 (3.2%)	102 (25.5%)
Middle educational level	932 (29.0%)	681 (27.5%)	68 (21.9%)	169 (42.2%)
High educational level	2,085 (64.9%)	1,709 (69.1%)	232 (74.8%)	129 (32.2%)
Missing	67	8	2	16
Comorbidities				
No comorbidities	1,698 (52.3%)	1,267 (51.4%)	158 (50.8%)	236 (59.3%)
Comorbidities	1,547 (47.7%)	1,199 (48.6%)	153 (49.2%)	162 (40.7%)
Missing	36	15	1	18
Financial hardship				
Yes	609 (18.9%)	357 (14.5%)	72 (23.5%)	169 (42.9%)
No	2,614 (81.1%)	2,113 (85.5%)	234 (76.5%)	225 (57.1%)
Missing	58	11	6	22
Vaccinated				
Yes	1,656 (50.5%)	1,143 (46.1%)	158 (50.6%)	314 (75.5%)
No	1,625 (49.5%)	1,338 (53.9%)	154 (49.4%)	102 (24.5%)

### COVID-19 vaccine acceptance and vaccine uptake

**Figure 2:**
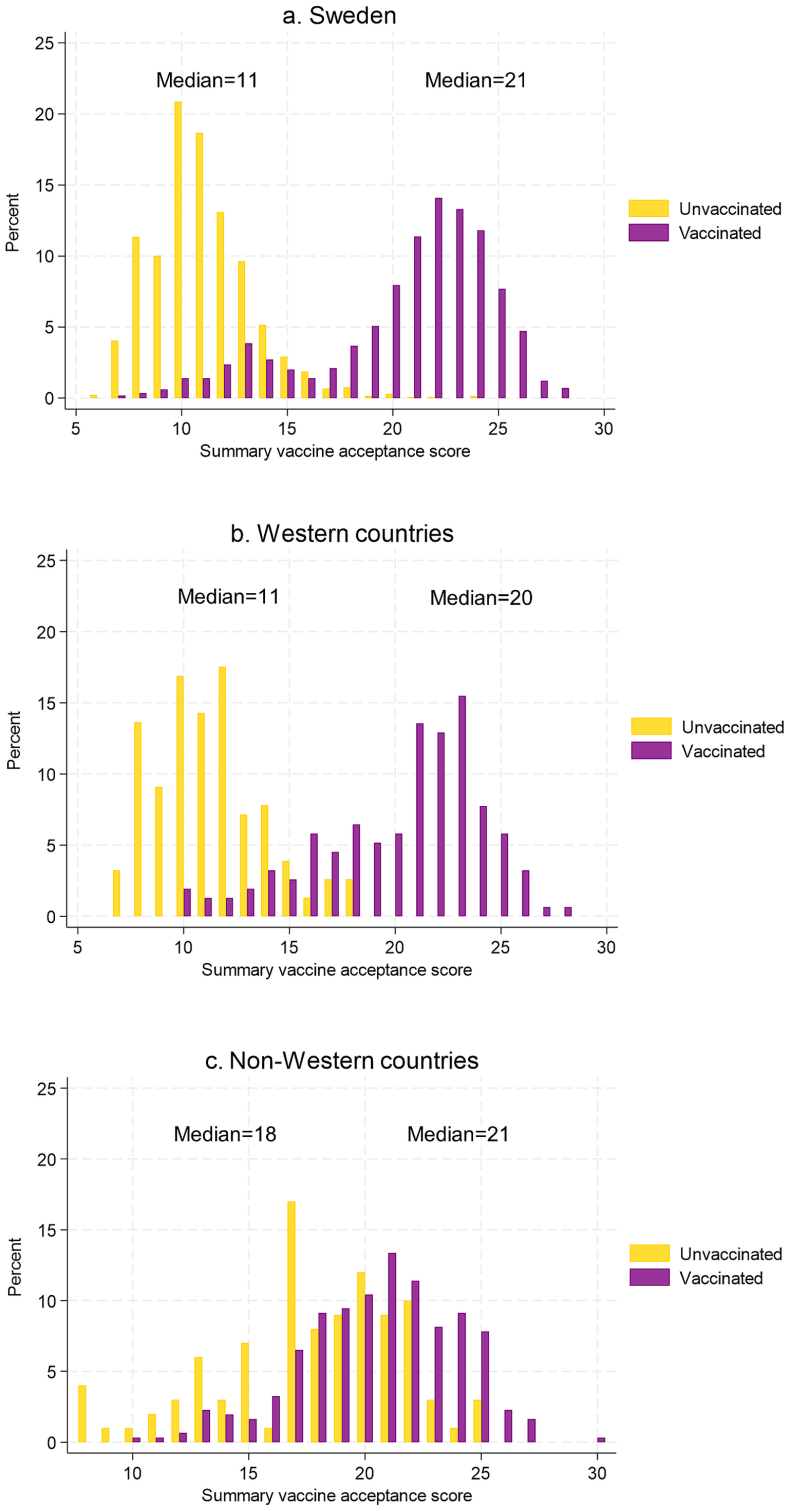
The weighted distribution of vaccine acceptance score for all domains combined (score range 6–30) by vaccination status stratified by region groups (**a**. Sweden; **b**. Western countries; and **c**. non-Western countries) and the respective median domain score by vaccination status.

Vaccine acceptance scores for all domains (range 6–30) by vaccination status showed higher vaccine acceptance among vaccinated than among unvaccinated individuals in all three region groups (Figures 2b,c). There was a clear separation of vaccine acceptance score by vaccination status among individuals from Sweden and Western countries (Figures 2a,b). By contrast, a larger overlap between vaccination status and scores was observed among individuals from non-Western countries, where unvaccinated individuals scored relatively high compared to the other groups, showcasing subtle rather than distinct differences in vaccine acceptance and uptake (Figure 2c). Similar patterns and differences across the region groups were also generally observed for each domain separately (Supplementary Table 5).

In the adjusted linear regression model (Supplementary Table 6; Model 1), vaccinated individuals had 8.8 (95% CI: 8.5 to 9.0) points higher acceptance score compared with unvaccinated individuals. Individuals from non-Western countries had, on average, 2.1 (95% CI: 1.8 to 2.7) points higher vaccine acceptance scores compared to individuals from Sweden after adjustments, including vaccination status. A differential association between vaccination status and vaccine acceptance, depending on the region group, was observed in the interaction model after adjustments (Supplementary Table 6; Model 1). Among individuals from Sweden and Western countries, vaccinated had 9.5 (95% CI: 9.2 to 9.7, *p* < 0.001) and 8.9 (95% CI: 8.1 to 9.7; 9.5–0.6) points, respectively, higher vaccine acceptance score on average, compared with only 2.8 (9.5–6.7 = 2.8, 95% CI: 2.0 to 3.6) higher average score among vaccinated from non-Western countries.

The general patterns of vaccinated individuals from Sweden and Western countries being more vaccine acceptant than unvaccinated individuals, and individuals of non-Western countries, irrespective of vaccination status, being vaccine acceptant, were also illustrated in the heatmap showcasing mean scores on the ordinal scale of the items within each domain (Table 2). Vaccinated individuals from Sweden and Western countries agreed more with questions reflecting vaccine acceptance, while unvaccinated individuals agreed more with questions reflecting vaccine hesitancy. For example, vaccinated individuals from Sweden and Western countries scored 2.1 and 2.2, respectively, on the item ‘*I don’t think COVID-19 vaccines are effective enough*’, while corresponding unvaccinated groups scored 3.8 and 3.8, respectively. The mean scores were generally similar across vaccinated and unvaccinated individuals from non-Western countries, generally leaning towards agreeing responses. For example, vaccinated individuals scored 3.1, and unvaccinated individuals 2.7 for the item ‘*I am concerned about infecting someone close to me with COVID-19*’. However, there were exceptions for these patterns, where both vaccinated and unvaccinated scored high on items indicating vaccine hesitancy as well, e.g., a mean score of 3.1 among vaccinated and 3.3 among unvaccinated for the item *‘I have a fear of vaccine side effects’*. Similarly, both vaccinated and unvaccinated individuals from Sweden, Western countries and non-Western countries, disagreed more with items included in the domain ‘descriptive norms’. The corresponding OR for agreeing responses and the associations with vaccine uptake in unadjusted and adjusted models were reported in the Supplementary material (Supplementary Table 1).

**Table 2:** Heatmap with the mean score on the ordinal scale for each question within each domain among vaccinated and unvaccinated stratified by region groups, grouped as Sweden, Western countries, and non-Western countries.

	Sweden		Western countries		Non-Western countries	
	Vaccinated	Unvaccinated	Vaccinated	Unvaccinated	Vaccinated	Unvaccinated
Trust in vaccines						
Trust						
I have received other vaccinations before and have good experience with it	3,6	2,7	3,4	2,6	3,1	2,6
Distrust						
I have seen or heard bad things about COVID-19 vaccine	2,8	3,8	2,9	3,6	3,2	3,3
Pharmaceutical companies make too much money developing vaccines	2,6	3,8	2,9	3,8	3,1	3,1
I don’t think COVID-19 vaccines are effective enough	2,1	3,8	2,2	3,8	2,7	3,1
Anyone who is vaccinated can be tracked and monitored by the authorities	1,3	2,3	1,4	2,5	2	2,1
Vaccine can make it easier to get infected with COVID-19	1,4	3,2	1,5	3,1	2,3	2,8
Benefit–risk balance						
Benefit						
I am concerned about infecting someone close to me with COVID-19	2,6	1,6	3,0	1,8	3,1	2,7
I’m afraid of becoming seriously ill with COVID-19	2,5	1,3	2,7	1,4	2,8	2,3
Risk						
I have a fear of vaccine side effects	2,2	3,6	2,3	3,6	3,1	3,3
I can stay healthy in other ways and therefore do not need to be vaccinated	1,8	3,7	1,9	3,7	2,7	3,3
Trust in institutions						
Confidence in researchers	3,4	2,3	3,3	2,4	3,1	2,8
Confidence in healthcare	3,2	2,1	3,1	2,1	3,2	2,8
Confidence in pharmaceutical companies	2,6	1,4	2,5	1,4	2,6	2,4
Confidence in politicians and policy makers	2,3	1,5	2,1	1,4	2,4	2,1
Injunctive norms						
It is important to stay at home if you have been infected with COVID-19	3,7	3,0	3,7	3,0	3,6	3,4
I dare to tell others about my opinions about vaccinations	3,7	3,3	3,4	3,2	3,1	3,0
It is good if vaccination certificates are required at larger public gatherings and events	2,8	1,1	2,8	1,2	3,0	2,5
The recommendations of the Public Health Agency of Sweden are important for my position on vaccinations	3,1	1,2	2,9	1,3	3,1	2,5
Descriptive norms						
In the context I live in, it is important to get vaccinated	3,2	1,3	3,0	1,3	3,1	2,2
Important that others around me also get vaccinated	2,7	1,1	2,6	1,1	2,9	2,3
There are people among my friends, in my family or relatives who are important to my decision regarding vaccination	2,4	1,5	2,3	1,5	2,8	2,4
There are people within associations or communities who are important for my decision regarding vaccination	1,5	1,2	1,7	1,3	2,2	1,9
Confidence in social media	1,3	1,4	1,4	1,3	2,1	2,1
There are people or groups on social media that are important to my decision regarding vaccination	1,3	1,5	1,4	1,3	2,0	2,1
Accessibility						
Important that I receive information about the vaccination in my language	3,3	2,8	2,7	2,3	3,4	3,2
Important that there are short waiting times for vaccination	3,0	1,6	3,1	1,7	3,4	3,0
Important that there are drop-in times for vaccinations so you do not have to book an appointment	2,5	1,6	2,6	1,8	3,1	3,0
Important that I receive a call for vaccination from my healthcare provider without me having to do anything	2,1	1,2	2,3	1,4	2,9	2,6
Important that you do not have to use BankID when booking an appointment for vaccination	1,5	1,9	1,8	1,7	2,6	2,6

The mean score is on a scale ranging from 1 to 4 (1 = Strongly disagreeing represented by the colour red; 2 = Disagreeing represented by the colour yellow; 3 = Agreeing represented by the colour light green; 4 = Strongly agreeing represented by the colour dark green).

### Associations between COVID-19 vaccine uptake and vaccine acceptance by region groups

**Figure 3:**
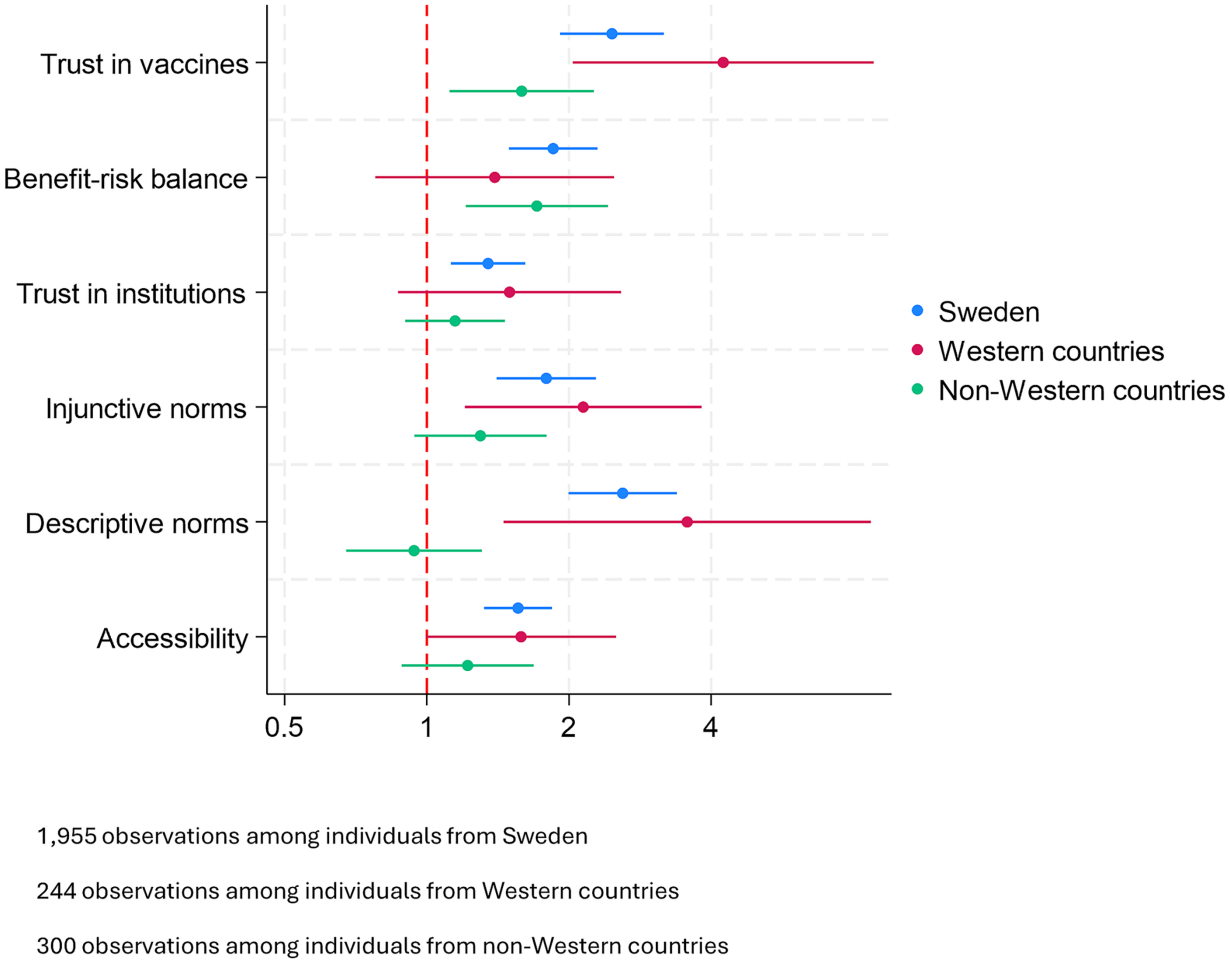
Associations between vaccine uptake and six domains in multidomain models stratified by region groups. Odds ratios (OR) and 95% confidence intervals (CI), adjusted by age, sex/gender, financial hardships, education, civil status, and comorbidities. The number of observations included in each stratified model.

After adjusting for sociodemographic factors, the associations between individual domains and vaccine uptake were generally stronger among individuals from Sweden and Western countries (Figure 3). Among individuals from Sweden, the strongest associations were observed for vaccine uptake and the domains ‘descriptive norms’ (OR = 2.6; 95% CI: 2.0–3.4) and ‘trust in vaccines’ (OR = 2.5; 95% CI: 1.9–3.2). Correspondingly, among individuals from Western countries, the domains ‘trust in vaccines’ (OR = 4.2; 95% CI: 2.0–8.8) and ‘descriptive norms’ (OR = 3.6; 95% CI: 1.5–8.7) had the strongest associations with vaccine uptake. These domains also showed the greatest differences in associations across region groups, with patterns diverging between individuals from Sweden or Western countries and those from non-Western countries. Additionally, in the model with individuals from non-Western countries, the conclusive associations observed were between vaccine uptake and the domains ‘benefit–risk balance’ (OR = 1.7; 95% CI: 1.2–2.4) and ‘trust in vaccines’ (OR = 1.6; 95% CI: 1.1–2.3). The differences in associations across region groups were relatively small concerning the domain ‘benefit–risk balance’. Lastly, the associations observed were stronger in the single domain models, but the patterns observed were generally similar to the multidomain models (Supplementary Table 7).

## Discussion

The results of this study, based on data from an anonymous survey adapted to a Swedish context, showed that associations between behavioural and social drivers of vaccination and actual vaccine uptake varied depending on region groups. In particular, the associational patterns were more similar among individuals from Sweden and Western countries compared to individuals from non-Western countries. In the latter group, the associations observed between the drivers and vaccine uptake were weaker, suggesting that behavioural and social drivers may differ in relevance and impact across region groups. These results were supported by the linear regression analysis, including the analysis of effect modification showing weaker associations between vaccine uptake and the summary vaccine acceptance score among individuals from non-Western countries compared to individuals from Sweden. This stresses the need for tailored public health efforts addressing low vaccine uptake, but also further research to better understand what influences vaccine uptake among individuals from non-Western countries living in Sweden, as research on this is limited in this particular context.

All domains had an independent association with vaccine uptake among individuals from Sweden, indicating that these domains captured different aspects of vaccine acceptance and hesitancy that influence vaccine uptake (35). The observed associations of social norms and ‘trust in vaccines’ among individuals from Sweden and Western countries corroborated the results in previous literature (36–38), including our previous study developing the vaccine acceptance scoring system in a Swedish population (33). Thus, future efforts promoting vaccine uptake should consider behavioural and social drivers of vaccination, including aspects of mistrust in vaccines and social norms varying across different contexts. Among individuals from non-Western countries, the differences between vaccinated and unvaccinated individuals were less pronounced in relation to vaccine acceptance, indicating that both unvaccinated and vaccinated individuals from non-Western countries were moderately positive towards vaccines. Similar to the latter finding, in a report by the Swedish Public Health Agency, it was also observed that immigrants in Sweden, born in Southwest Asia/Middle East and Africa, were generally vaccine acceptant. Despite this, the vaccine uptake was still lower among these groups than in the general population (39). These findings indicate that vaccine uptake in this group may be improved through limited, targeted interventions, whereas other region groups, with more varied attitudes towards vaccination, might require broader, more resource-intensive strategies. Nonetheless, the results of this study suggest that the BeSD framework, as operationalised in this study, may not sufficiently capture the factors that influenced vaccine uptake among individuals from non-Western countries. Somewhat similar results have been seen in other studies conducted in Canada, where results of no association between vaccine hesitancy and certain ethnic/racial groups were observed (40–42).

Several plausible explanations could underlie the weaker associations observed among individuals from non-Western countries. On the one hand, other factors that influenced the decision to vaccinate may not have been captured by the BeSD framework. Structural and historical factors have been observed to play an important role in healthcare access (26) as well as vaccine uptake and hesitancy among marginalised communities (43–45). Despite this, similar to what has been highlighted previously (45), these factors were not sufficiently considered. Thus, this framework poses a limitation to capturing multi-level factors influencing vaccine uptake. On the other hand, the domain ‘accessibility’ included important contextual factors potentially hindering access to healthcare and vaccination for minoritised individuals (43, 46), and might have interacted with the other domains, diluting the associations. Another potential reason for the weaker observed associations may be related to the domains, including the domain ‘accessibility’, which reflected *perceived* barriers, and not necessarily *experienced* barriers, where the latter may still play a role in shaping vaccine hesitancy and acceptance. Many unvaccinated individuals responded ‘no opinion’ to items within this domain and were thus not given a score for the ‘accessibility’ domain. This could have influenced our results, and the potential influence of accessibility and the other domains on vaccine uptake may not have been captured. Thus, questionnaire items could be rephrased to capture experienced rather than perceived inaccessibility to vaccination services in future research. This applies to the other domains as well, where perceptions could differ from experiences. Further research would be needed to better understand existing barriers to vaccine uptake among individuals from non-Western countries, whether behavioural and social drivers of vaccination are relevant, and if so, how measurements aiming to capture vaccine hesitancy and acceptance among individuals from non-Western countries may be better operationalised.

### Policy implications

Promoting vaccine uptake by addressing vaccine hesitancy in groups with differing attitudes might constitute a challenge for designing successful and relevant interventions concerning identifying, reaching, and engaging hesitant individuals (47, 48). By focusing on similarities, the domains ‘accessibility’ and ‘trust in institutions’, where unvaccinated individuals expressed moderate rather than strong disagreement, could be an initial target to establish common ground (47, 49), potentially facilitating further empathic interventions. Among individuals from non-Western countries, similar attitudes were seen among vaccinated and unvaccinated individuals. In this context, interventions applied to the whole group could be more efficient and potentially impactful in promoting vaccine uptake, as fewer additional efforts would be required to identify unvaccinated individuals. Based on our results, addressing aspects of distrust in the vaccine, as well as highlighting the potential benefits of the vaccine, may influence vaccine acceptance and, in turn, promote a higher vaccine uptake. Although the distrust of vaccines and the perceptions of risks related to the vaccine were experienced at the individual level, it reflects concerns rooted in multilevel factors, requiring efforts addressing individual as well as contextual factors. Nonetheless, further research and prioritisation from policymakers would be required to better understand the reasons for low vaccine uptake and how to effectively promote vaccine uptake among individuals from non-Western countries. The research on this is limited, especially in Sweden. Thus, facilitating and endorsing further investigations is a necessary step to prevent health disparities and ensure equal health in the Swedish population.

### Limitations

The results of this study should be understood in light of its strengths and limitations. We used non-probability sampling techniques, namely volunteer and purposive sampling, as a response to the low participation rate of various population groups in our previous study (33). There are several aspects to consider. Our sample consisted of slightly older individuals (50–64 years), more women, highly educated, and in partnership (‘not single’). Although our sample consisted of slightly older individuals, the oldest age group was not adequately represented, which may also influence the results of this study, considering that the groups not sufficiently represented could have other perceptions and experiences of disease risks and barriers to vaccination during the pandemic. Additionally, in Sweden, ‘cohabitee’ relationships (*samboförhållande*) are common, but not registered as a relationship in register data. Meanwhile, our survey data captured this type of relationship. Therefore, the observed differences between our sample and the population subgroups of Skåne may be due to this discrepancy. Also, there were observed differences among the region groups. Individuals from Sweden and Western countries were to a higher degree unvaccinated and more highly educated compared to individuals from non-Western countries. The aforementioned differences could have been due to a varying recruitment strategy in different years, as individuals from Sweden and Western countries primarily responded through the digitally disseminated questionnaire during 2023 (volunteer sampling), whereas individuals from non-Western countries were more likely to complete the paper version in 2024, facilitated by outreach from authors LB, MH, and a project assistant (purposive sampling). This may have introduced bias. For instance, attitudes could change over time, and additionally, the volunteer sampling may have reached groups that were more interested in the topic and had higher engagement with digital resources compared to the groups reached using purposive sampling. In our linear regression analysis, we adjusted for sociodemographic information and vaccination uptake. The results were consistent with the patterns observed in the stratified analysis, where the associations between vaccinated and unvaccinated individuals were weaker among individuals from non-Western countries compared to individuals born in Sweden. Nevertheless, inferring study results to the population subgroups of Skåne should be done with care, although this recruitment approach enabled the inclusion of groups that are often underrepresented in research.

Additionally, the categorisation of region groups as Western/non-Western was rudimentary and has been used as a form of othering rooted in power relations, such as colonialism and historical patterns of exploitation contributing to global inequalities (50, 51). Using these categories risks reproducing categories of populations, creating problematic generalisations. However, critically using terms based on socially constructed classifications can serve as analytical tools to expose systemic and structural inequalities and the historically embedded power relations that produce patterned disadvantages across multiple domains of social life, as seen internationally and within contemporary European states, where immigrants from non-Western countries often experience worse outcomes (51), including health outcomes (10, 52).

Furthermore, we offered the questionnaire in several languages, aiming to eliminate potential issues in language proficiency. However, potential bias related to the questionnaire format cannot be ruled out, as differences in literacy levels may have influenced how individuals interpreted and responded to specific response options (53).

## Conclusion

This study contributed to the knowledge of drivers and barriers to vaccination in region groups. The findings highlighted that multiple factors influenced vaccine uptake, although there were significant differences in relevance and impact of behavioural and social factors across different region groups. The associations between vaccine uptake and vaccine hesitancy/acceptance were stronger among individuals from Sweden and Western countries than among individuals from non-Western countries. For some domains, the strongest associations were observed among individuals from Western countries, reflecting differences in the potential drivers of vaccine uptake. Future steps require addressing these factors to promote vaccine uptake, and further research is needed to better understand what influences vaccine uptake among individuals from non-Western countries as this is sparse in Sweden, limiting our understanding of why we observe a lower vaccine uptake in this specific context.

## Data Availability

The datasets presented in this article are not readily available because data are available upon reasonable request but may require a new ethical approval. Requests to access the datasets should be directed to Mariam Hassan, mariam.hassan@med.lu.se.
