# Supplementary material for: Differences in COVID-19 vaccine acceptance and uptake according to region of birth: findings from a cross-sectional survey in Sweden
Source: Front Public Health. 2026 Jun 24;14:1861648. doi: 10.3389/fpubh.2026.1861648 (PMC13341694; doi:10.3389/fpubh.2026.1861648)
Supplement: Supplementary file 1 [file Table_1.pdf]

Supplementary materials

Table S1 Odds Ratios (OR) by items included in each domain stratified by region groups. Unadjusted models and adjusted models by age, sex/gender, financial hardships, educational level, civil status, and comorbidities. For each question, it is indicated whether agreeing or disagreeing responses to the question yield 1 point (p) in the vaccine acceptance scoring

	**Responses yielding 1p**	**Sweden**		**Western countries**		**Non-Western countries**	
**Trust in vaccines**		Unadjusted	Adjusted	Unadjusted	Adjusted	Unadjusted	Adjusted
I have had other vaccinations in the past and have good experience of that	Agreeing responses	5.9 (4.7–7.3)	6.4 (5.0–8.2)	4.0 (2.3–6.8)	3.6 (2.0–6.5)	3.9 (2.4–6.3)	4.3 (2.5–7.5)
Observations		2,473	2,338	308	288	396	336
Pharmaceutical companies make too much money developing vaccines	Disagreeing responses	40.0 (27.3–58.5)	43.6 (28.6–66.5)	38.4 (9.1–161.2)	84.1 (11.1–639.1)	0.9 (0.5–1.6)	1.0 (0.5–1.9)
Observations		2,454	2,319	306	286	380	320
I've seen or heard bad things about COVID-19 vaccines	Disagreeing responses	17.2 (12.4–23.7)	16.5 (11.6–23.4)	5.5 (2.8–10.6)	4.9 (2.4–10.2)	1.2 (0.6–2.2)	1.1 (0.5–2.2)
Observations		2,473	2,336	309	289	395	333
Vaccines can make it easier to get infected with COVID-19	Disagreeing responses	38.1 (29.9–48.6)	40.9 (31.1–53.9)	15.4 (8.6–27.5)	18.5 (9.4–36.6)	2.7 (1.7–4.3)	2.9 (1.6–4.9)
Observations		2,453	2,321	305	286	393	331
I don't think the vaccines against COVID-19 are effective enough	Disagreeing responses	38.9 (30.0–50.3)	36.7 (27.8–48.4)	33.5 (15.8–71.2)	36.7 (15.6–86.2)	2.0 (1.2–3.4)	2.0 (1.1–3.6)
Observations		2,472	2,338	308	288	381	323
The vaccinated person can be tracked and monitored by the authorities	Disagreeing responses	9.0 (7.1–11.6)	10.6 (8.1–14.0)	7.0 (3.8–12.8)	9.6 (4.6–20.2)	1.2 (0.7–1.9)	1.2 (0.7–2.1)
Observations		2,452	2,320	306	287	385	326
**Benefit-risk balance**							

I can stay healthy in other ways and therefore do not need to be vaccinated	Disagreeing responses	66.2 (49.7–88.1)	57.8 (42.9–77.9)	27.8 (14.3–54.3)	33.5 (15.3–73.2)	4.7 (2.4–9.5)	4.5 (2.0–10.1)
Observations		2,474	2,336	307	287	392	329
I am concerned about infecting someone close to me with COVID-19	Agreeing responses	8.5 (6.9–10.3)	7.4 (6.0–9.2)	6.6 (4.0–10.8)	5.5 (3.1–9.8)	1.8 (1.1–2.9)	1.9 (1.1–3.3)
Observations		2,473	2,337	307	287	393	333
I feel fear of suffering side effects of vaccines	Disagreeing responses	23.4 (18.6–29.4)	25.0 (19.4–32.4)	15.2 (8.3–28.1)	18.9 (9.3–38.3)	1.0 (0.6–1.8)	1.2 (0.6–2.4)
Observations		2,472	2,336	309	288	397	337
I am afraid of getting seriously ill from COVID-19	Agreeing responses	27.9 (19.9–39.2)	22.1 (15.4–31.7)	14.4 (7.4–28.0)	11.7 (5.6–24.5)	3.1 (1.9–4.9)	3.2 (1.8–5.4)
Observations		2,480	2,343	309	288	402	337
**Trust in institutions**							
Confidence in healthcare	Agreeing responses	15.0 (12.3–18.3)	15.7 (12.6–19.7)	13.0 (7.5–22.3)	16.4 (8.6–31.3)	2.3 (1.4–4.0)	3.2 (1.8–6.0)
Observations		2,452	2,318	308	287	373	313
Confidence in politicians	Agreeing responses	19.6 (14.2–27.0)	19.9 (14.1–28.0)	11.5 (4.4–30.0)	10.9 (4.0–30.0)	1.6 (1.0–2.8)	2.0 (1.1–3.6)
Observations		2,447	2,314	303	283	331	270
Confidence in researchers	Agreeing responses	17.4 (13.7–22.1)	16.8 (12.9–21.8)	10.4 (5.7–19.1)	10.0 (5.1–19.6)	2.1 (1.2–3.5)	1.8 (1.0–3.4)
Observations		2,388	2,261	299	279	349	297
Confidence in pharmaceutical companies	Agreeing responses	68.1 (47.0–98.7)	61.8 (42.2–90.7)	30.5 (12.7–73.4)	28.5 (11.4–71.0)	1.4 (0.9–2.3)	1.5 (0.8–2.5)
Observations		2,410	2,281	303	283	343	292
**Injunctive norms**							
The recommendations of the Public Health Agency of Sweden are important for my position on vaccination	Agreeing responses	84.9 (62.2–116.0)	80.6 (57.8–112.3)	35.2 (16.9–73.0)	40.9 (18.3–95.8)	3.2 (1.9–5.2)	3.3 (1.9–5.7)
Observations		2,477	2,340	305	287	388	330
I dare to tell others about my opinions about vaccinations	Agreeing responses	4.5 (3.4–6.0)	4.6 (3.3–6.2)	1.8 (1.0–3.2)	2.0 (1.0–4.1)	1.5 (0.9–2.5)	1.3 (0.7–2.3)

Observations		2,475	2,338	309	288	387	324
It is important to stay at home if you have been infected with COVID-19	Agreeing responses	6.0 (4.6–7.9)	4.8 (3.6–6.5)	6.1 (2.9–12.6)	5.3 (2.3–11.9)	1.7 (0.8–3.7)	2.2 (0.9–4.9)
Observations		2,466	2,331	309	287	399	333
It is good if vaccination certificates are required at larger public gatherings and events	Agreeing responses	107.9 (67.6–172.4)	111.5 (66.6–186.6)	27.4 (12.6–60.0)	45.7 (16.9–124.0)	2.7 (1.7–4.4)	3.0 (1.8–5.2)
Observations		2,473	2,335	306	285	395	333
**Descriptive norms**							
Important that others around me also get vaccinated	Agreeing responses	168.4 (89.3–317.5)	182.2 (89.4–371.4)	54.6 (16.6–179.6)	41.8 (12.4–141.4)	2.1 (1.3– 3.5)	3.0 (1.7–5.5)
Observations		2,274	2,160	278	261	352	298
Confidence in social media influencers	Agreeing responses	0.4 (0.2–0.7)	0.4 (0.2–0.8)	1.9 (0.6–6.4)	1.3 (0.3–5.6)	0.9 (0.5–1.5)	1.0 (0.6–1.9)
Observations		2,326	2,200	286	268	333	282
In the context I live in, it is important to get vaccinated	Agreeing responses	43.3 (33.8–55.6)	39.3 (30.1–51.2)	40.7 (19.5–84.9)	39.9 (18.0–88.4)	3.4 (2.1–5.5)	3.4 (2.0–5.9)
Observations		2,473	2,338	307	288	390	330
There are people or groups on social media that are important to my decision regarding vaccination	Agreeing responses	0.3 (0.2–0.4)	0.4 (0.3–0.5)	0.9 (0.4–2.1)	0.9 (0.3–2.4)	0.6 (0.4–1.0)	0.6 (0.3–1.0)
Observations		2,475	2,339	308	288	389	333
There are people within associations or communities who are important for my decision regarding vaccination	Agreeing responses	3.8 (2.8–5.1)	3.4 (2.4–4.8)	2.9 (1.4–6.3)	3.2 (1.3–7.9)	1.4 (0.8–2.3)	1.3 (0.8–2.3)
Observations		2,475	2,338	306	287	390	331
There are people among my friends and family who are important in my decision regarding vaccination	Agreeing responses	6.2 (5.1–7.6)	6.0 (4.8–7.4)	5.0 (2.8–8.8)	4.6 (2.4–8.6)	1.4 (0.9–2.3)	1.5 (0.9–2.6)
Observations		2,477	2,340	309	289	393	332
**Accessibility**							

Important that I receive information about the vaccination in my language	Agreeing responses	2.4 (1.9–2.9)	2.1 (1.6–2.6)	1.5 (0.9–2.5)	1.4 (0.8–2.5)	1.4 (0.7–2.6)	1.5 (0.7–3.2)
Observations		1,781	1,689	248	232	378	315
Important that I receive a call for vaccination from my healthcare provider without me having to do anything	Agreeing responses	15.4 (10.6–22.4)	14.1 (9.4–21.2)	6.8 (3.3–14.1)	5.9 (2.6–13.3)	1.5 (0.9–2.5)	1.6 (0.9–2.8)
Observations		2,021	1,912	253	239	360	306
Important that there are drop in times for vaccination so you do not have to book an appointment	Agreeing responses	5.2 (4.1–6.5)	4.6 (3.6–5.9)	3.0 (1.7–5.5)	2.7 (1.4–5.2)	1.4 (0.8–2.6)	1.9 (0.9–3.7)
Observations		1,812	1,713	231	216	354	300
Important that you do not have to use BankID when booking an appointment for vaccination	Agreeing responses	0.4 (0.3–0.5)	0.3 (0.3–0.4)	1.2 (0.6–2.2)	0.8 (0.4–1.8)	0.8 (0.5–1.4)	0.8 (0.4–1.5)
Observations		1,861	1,758	220	207	347	294
Important that there are short waiting times for vaccination	Agreeing responses	13.5 (10.6–17.2)	12.2 (9.4–15.8)	17.4 (8.8–34.4)	16.1 (7.5–34.5)	1.7 (0.9–3.3)	2.1 (0.9–4.5)
Observations		1,773	1,679	230	214	364	306

Table S2 Cronbach's alpha for the internal consistency of the domains separately and aggregated in a summary variable for the survey sample.

**Domain**	**Cronbach's Alpha**
Trust in vaccines	0.76
Benefit-risk balance	0.74
Trust in institutions	0.79
Injunctive norms	0.60
Descriptive norms	0.63
Accessibility	0.66
All domains	0.87

Table S3 Descriptive characteristics of the participants in the study and the underlying adult population of Skåne using both register data and survey data

	**Sweden** (n=2,481)	**Swedish born adult population in Skåne** (N=843,372)	**Western countries** (n=312)	**Western countries in the population of Skåne** (N=150,870)	**Non-Western countries** (n=416)	**Non-Western countries in the population of Skåne**(N=63,547)
**Age**						
18–49	972 (40.0%)	389,635 (46.2%)	137 (45.1%)	75,375 (50.0%)	238 (66.3%)	44,976 (68.1%)
50–64	975 (40.2%)	189,145 (22.4%)	125 (41.1%)	36,899 (24.5%)	107 (29.8%)	13,682 (21.0%)
65+	480 (19.8%)	264,592 (31.4%)	42 (13.8%)	38,596 (25.6%)	14 (3.9%)	4,889 (10.9)
Missing	54		8		57	
**Gender/sex**						
Women	1,723 (71.4%)	535,551 (50.2%)	227 (73.9%)	81,628 (50.8%)	292 (71.6%)	28,257 (44.5%)
Men	690 (28.6%)	531,433 (49.8%)	80 (26.1%)	79,090 (49.2%)	116 (28.4%)	35,290 (55.5%)
Missing	68		5		8	
**Civil status**						
Single	738 (29.9%)	740,721 (69.4%)	87 (28.2%)	78,843 (52.3%)	126 (31.9%)	30,305 (47.7%)
Not single	1,732 (70.1%)	326,263 (38.7%)	221 (71.8%)	72,027 (47.74%)	269 (68.1%)	33,242 (52.3%)
Missing	11		4		21	
**Education**						
Low educational level	83 (3.4%)	171,899 (20.4%)	10 (3.2%)	41,659 (27.6%)	102 (25.5%)	27,932 (44.0%)
Middle educational level	681 (27.5%)	353,611 (41.9%)	68 (21.9%)	55,175 (36.6%)	169 (42.2%)	17,872 (28.1%)
High educational level	1,709 (69.1%)	317,862 (37.7%)	232 (74.8%)	54,036 (35.8%)	129 (32.2%)	17,743 (27.9%)
Missing	8		2		16	
**Vaccination status**						
Vaccinated	1,143 (46.1%)	756,497 (89.7%)	158 (50.6%)	100,885 (66.9%)	314 (75.5%)	42,391 (66.7%)
Unvaccinated	1,338 (53.9%)	86,875 (10.3%)	154 (49.4%)	49,985 (33.1%)	102 (24.5%)	21,156 (33.3%)

Table S4 Descriptive characteristics of the participants by region groups and vaccination status

	**Sweden (n=2,481)**		**Western countries (n=312)**		**non-Western countries (n=416)**	
**Mode of administration of questionnaires by year**	Vaccinated (n=1,143)	Unvaccinated (1,338)	Vaccinated (n=154)	Unvaccinated (n=158)	Vaccinated (n=314)	Unvaccinated (n=102)
**Year 2023**						
Digital	1,119 (99.7%)	1,331 (100.0%)	121 (100.0%)	144 (100.0%)	37 (36.8%)	10 (41.7%)
Paper form	3 (0.3%)	0 (0.0%)	0 (0.0%)	0 (0.0%)	61 (62.2%)	14 (58.3%)
**Year 2024**						
Digital	1 (4.8%)	0 (0.0%)	1 (2.7%)	0 (0.0%)	7 (3.2%)	1 (1.3%)
Paper form	20 (95.2%)	7 (100.0%)	36 (97.3%)	10 (100.0%)	209 (96.8%)	77 (98.7%)
**Age**						
18-49	404 (36.1%)	568 (43.4%)	77 (50.0%)	60 (40.0%)	188 (66.2%)	50 (66.7%)
50-64	417 (37.3%)	558 (42.6%)	60 (39.0%)	65 (43.3%)	84 (29.6%)	23 (30.7%)
65+	297 (26.6%)	183 (14.0%)	17 (11.0%)	25 (16.7%)	12 (4.2%)	2 (2.7%)
Missing	28	26	6	2	50	8
**Sex/gender**						
Women	875 (78.5%)	848 (65.3%)	120 (77.9%)	107 (69.9%)	227 (73.5%)	65 (65.7%)
Men	240 (21.5%)	450 (34.7%)	34 (22.1%)	46 (30.1%)	82 (26.5%)	34 (34.3%)
Missing	28	40	4	1	5	3
**Civil status**						
Single	355 (31.2%)	383 (28.7%)	37 (24.0%)	50 (32.5%)	95 (31.9%)	31 (32.0%)
Not single	781 (68.8%)	951 (71.3%)	117 (76.0%)	104 (67.5%)	203 (68.1%)	66 (68.0%)
Missing	7	4	4	0	16	5
**Education**						
Low educational level	40 (3.5%)	43 (3.2%)	5 (3.2%)	5 (3.2%)	79 (26.1%)	23 (23.7%)
Middle educational level	243 (21.3%)	438 (32.9%)	33 (21.2%)	35 (22.7%)	122 (40.3%)	47 (48.5%)
High educational level	857 (75.2%)	852 (63.9%)	118 (75.6%)	114 (74.0%)	102 (33.7%)	27 (27.8%)
Missing	3	5	2	0	11	5
**Comorbidities**						
No comorbidities	399 (35.2%)	868 (65.2%)	56 (35.4%)	102 (66.7%)	168 (56.6%)	68 (67.3%)
Comorbidities	735 (64.8%)	464 (34.8%)	102 (64.6%)	51 (33.3%)	129 (43.4%)	33 (32.7%)
Missing	9	6	0	1	17	1
**Financial hardship**						
Yes	189 (16.6%)	168 (12.6%)	42 (27.3%)	30 (19.7%)	130 (43.2%)	39 (41.9%)
No	948 (83.4%)	1,165 (87.4%)	112 (72.7%)	122 (80.3%)	171 (56.8%)	54 (58.1%)
Missing	6	5	4	2	13	9

**Table S5** The distribution of vaccine acceptance score by region groups and vaccination status						
**Domains**	**Sweden**		**Western countries**		**Non-Western countries**	
**Trust in vaccines**	Vaccinated	Unvaccinated	Vaccinated	Unvaccinated	Vaccinated	Unvaccinated
1	18 (1.6%)	226 (16.9%)	2 (1.3%)	29 (18.8%)	11 (3.6%)	12 (12.4%)
2	120 (10.5%)	901 (67.3%)	20 (13.0%)	89 (57.8%)	101 (33.3%)	46 (47.4%)
3	134 (11.7%)	167 (12.5%)	25 (16.2%)	29 (18.8%)	92 (30.4%)	22 (22.7%)
4	632 (55.3%)	42 (3.1%)	91 (59.1%)	7 (4.5%)	87 (28.7%)	16 (16.5%)
5	239 (20.9%)	2 (0.1%)	16 (10.4%)	0 (0.0%)	12 (4.0%)	1 (1.0%)
Missing	0	0	4	0	11	5
**Benefit-risk balance**						
1	130 (11.4%)	1030 (77.0%)	13 (8.4%)	99 (64.3%)	31 (10.2%)	25 (25.0%)
2	108 (9.4%)	245 (18.3%)	15 (9.7%)	38 (24.7%)	76 (24.9%)	33 (33.0%)
3	300 (26.2%)	52 (3.9%)	39 (25.3%)	12 (7.8%)	113 (37.0%)	34 (34.0%)
4	321 (28.1%)	10 (0.7%)	43 (27.9%)	5 (3.2%)	65 (21.3%)	8 (8.0%)
5	284 (24.8%)	1 (0.1%)	44 (28.6%)	0 (0.0%)	20 (6.6%)	0 (0.0%)
Missing	0	0	4	0	9	2
**Trust in institutions**						
1	73 (6.4%)	738 (55.6%)	6 (3.9%)	80 (52.3%)	19 (6.7%)	10 (11.0%)
2	100 (8.8%)	346 (26.1%)	23 (14.8%)	44 (28.8%)	48 (16.8%)	26 (28.6%)
3	211 (18.5%)	183 (13.8%)	42 (27.1%)	22 (14.4%)	57 (20.0%)	14 (15.4%)
4	358 (31.4%)	45 (3.4%)	49 (31.6%)	5 (3.3%)	63 (22.1%)	20 (22.0%)
5	397 (34.9%)	15 (1.1%)	35 (22.6%)	2 (1.3%)	98 (34.4%)	21 (23.1%)
Missing	4	11	3	1	29	11
**Injunctive norms**						
1	11 (1.0%)	42 (3.1%)	2 (1.3%)	7 (4.5%)	3 (1.0%)	1 (1.0%)
2	64 (5.6%)	540 (40.4%)	11 (7.1%)	60 (39.0%)	14 (4.6%)	16 (16.3%)
3	155 (13.6%)	712 (53.3%)	25 (16.1%)	74 (48.1%)	40 (13.1%)	31 (31.6%)
4	294 (25.7%)	38 (2.8%)	47 (30.3%)	12 (7.8%)	108 (35.3%)	21 (21.4%)
5	618 (54.1%)	4 (0.3%)	70 (45.2%)	1 (0.6%)	141 (46.1%)	29 (29.6%)
Missing	1	2	3	0	8	4
**Descriptive norms**						
1	111 (9.7%)	950 (71.0%)	21 (13.5%)	112 (72.7%)	15 (4.9%)	18 (18.4%)
2	631 (55.2%)	342 (25.6%)	76 (49.0%)	38 (24.7%)	95 (31.2%)	33 (33.7%)
3	286 (25.0%)	34 (2.5%)	40 (25.8%)	4 (2.6%)	92 (31.2%)	22 (22.4%)
4	110 (9.6%)	12 (0.9%)	18 (11.6%)	0 (0.0%)	77 (25.3%)	20 (20.4%)
5	5 (0.4%)	0 (0.0%)	0 (0.0%)	0 (0.0%)	22 (7.2%)	5 (5.1%)
Missing	0	0	3	0	10	4
**Accessibility**						
1	96 (8.5%)	333 (35.1%)	7 (4.6%)	44 (40.7%)	9 (3.0%)	6 (6.8%)
2	193 (17.2%)	270 (28.5%)	33 (21.6%)	31 (28.7%)	16 (5.2%)	8 (9.1%)
3	507 (45.1%)	247 (26.0%)	67 (43.8%)	18 (16.7%)	77 (25.2%)	20 (22.7%)
4	252 (22.4%)	73 (7.7%)	29 (19.0%)	10 (9.3%)	93 (30.5%)	20 (22.7%)
5	77 (6.8%)	26 (2.7%)	17 (11.1%)	5 (4.6%)	110 (36.1%)	34 (38.6%)
Missing	18	389	5	46	9	14

**Table S6** Multiple linear regression analysis of the total summary vaccine acceptance score, vaccination status, and region groups adjusted by sex/gender, age, educational level, civil status, financial hardships, and comorbidities. Interaction analysis presented in Model 2		
	**Model 1**	**Model 2**
**Region group**		
Sweden	Reference	Reference
Western countries	-0.1 (-0.5 to 0.3)	0.1 (-0.4 to 0.7)
Non-Western countries	2.1 (1.7 to 2.5)	6.9 (6.2 to 7.7)
**Vaccination status**		
Vaccinated	8.8 (8.5 to 9.0)	9.5 (9.2 to 9.7)
Unvaccinated	Reference	Reference
**Region group x Vaccination status**		
Sweden x Vaccinated	-	Reference
Western countries x Vaccinated	-	-0.6 (-1.4 to 0.2)
Non-Western countries x Vaccinated	-	-6.7 (-7.5 to -5.8)
Intercept	10.4 (10.1 to 10.7)	10.2 (9.9 to 10.5)
P-value for the interaction analysis: p<0.001		

Table S7 Associations between vaccine uptake and six domains in single domain models and multidomain models stratified by region groups. Odds ratios (OR) and 95% confidence intervals (CI), unadjusted and adjusted by age, sex/gender, financial hardships, education, civil status, and comorbidities

	**Unadjusted models**			**Adjusted models**		
**Single domain models**	Sweden Model 1a	Western countries Model 1b	non-Western countries Model 1c	Sweden Model 2a	Western countries Model 2b	non-Western countries Model 3c
Trust in vaccines	8.4 (7.2–9.7)	6.5 (4.5–9.5)	1.7 (1.3–2.3)	9.0 (7.7–10.7)	6.9 (4.5–10.5)	1.9 (1.4–2.6)*
Observations	2,481	308	400	2,343	288	338
Benefit-risk balance	6.4 (5.6–7.3)	4.8 (3.5–6.6)	1.9 (1.5–2.4)	6.3 (5.5–7.3)	5.3 (3.6–7.7)	1.9 (1.4–2.5)*
Observations	2,481	308	405	2,343	288	342
Trust in institutions	4.2 (3.8–4.6)	4.4 (3.2–6.0)	1.3 (1.1–1.5)	4.4 (3.9–4.9)	4.4 (3.1–6.1)	1.4 (1.1–1.7)*
Observations	2,466	308	376	2,331	288	318
Injunctive norms	9.2 (7.7–10.8)	5.5 (3.8–7.9)	1.8 (1.4–2.2)	8.8 (7.4–10.5)	6.5 (4.2–10.1)**	1.8 (1.4–2.4)*
Observations	2,478	309	404	2,340	288	339
Descriptive norms	9.2 (7.7–10.9)	9.0 (5.5–14.7)	1.4 (1.1–1.8)	8.9 (7.3–10.7)	10.2 (5.7–18.1)	1.5 (1.2–1.9)*
Observations	2,481	309	402	2,343	288	340
Accessibility	2.2 (2.0–2.4)	2.4 (1.8–3.1)	1.1 (0.9–1.4)	2.1 (1.9–2.3)	2.2 (1.6–2.9)	1.2 (0.9–1.6)*
Observations	2,074	261	393	1,961	244	329

	**Unadjusted models**			**Adjusted models**		
**Multidomain models**	Sweden Model 3a	Western countries Model 3b	non-Western countries Model 3c	Sweden Model 4a	Western countries Model 4b	non-Western countries Model 4c
Trust in vaccines	2.2 (1.8–2.8)	3.7 (1.9–6.9)	1.5 (1.1–2.1)	2.5 (1.9–3.2)	4.2 (2.0–8.8)	1.6 (1.1–2.3)
Benefit-risk balance	1.9 (1.5–2.3)	1.4 (0.9–2.3)	1.7 (1.3–2.3)	1.9 (1.5–2.3)	1.4 (0.8–2.5)	1.7 (1.2–2.4)
Trust in institutions	1.3 (1.1–1.5)	1.6 (1.0–2.6)	1.1 (0.9–1.3)	1.3 (1.1–1.6)	1.5 (0.9–2.6)	1.1 (0.9–1.5)
Injunctive norms	1.9 (1.5–2.3)	1.9 (1.1–3.2)	1.4 (1.0–1.8)	1.8 (1.4–2.3)	2.1 (1.2–3.8)	1.3 (0.9–1.8)
Descriptive norms	2.8 (2.2–3.6)	3.9 (1.8–8.4)	1.0 (0.7–1.3)	2.6 (2.0–3.4)	3.6 (1.5–8.7)	0.9 (0.7–1.3)
Accessibility	1.5 (1.3–1.8)	1.7 (1.1–2.7)	1.1 (0.8–1.4)	1.6 (1.3–1.8)	1.6 (1.0–2.5)	1.2 (0.9–1.7)
Observations	2,066	260	356	1,955	244	300

P-values for significant results from a separate analysis of interactions between region groups and the six domains separately adjusted by age, sex/gender, financial hardships, education, civil status and comorbidities *p<0.001; **p=0.03
